# Primary Closure vs T-Tube Drainage Following Open Choledochotomy for CBD (Common Bile Duct) Stones

**DOI:** 10.7759/cureus.35846

**Published:** 2023-03-06

**Authors:** Yousaf Jan, Musarrat Hussain, Zahid Aman, Neelma N

**Affiliations:** 1 Department of General Surgery, Hayatabad Medical Complex Peshawar, Peshawar, PAK

**Keywords:** cbd, t-tube, choledocholithiasis, primary closure, open choledocotomy

## Abstract

Background: Choledocholithiasis affects 10%-15% of individuals with gallbladder stones. Approximately 7%-15% of cholecystectomy patients develop common bile duct (CBD) stones. It occurs more frequently in older age groups. It may result in a complete or partial blockage of the bile duct and present as pancreatitis or cholangitis due to gallstones.

Objective: The aim of this study was to compare the outcomes of primary closure of duct and T-tube drainage after open choledochotomy and to evaluate the effectiveness and safety of primary closure.

Material and methods: From January 2018 to December 2021, this descriptive study was carried out in the surgical department of the Hayatabad Medical Complex in Peshawar. In total, 60 patients who had open choledocholithotomy after a failed endoscopic extraction were included in this study. Of these, 30 (50%) patients got primary duct closure after choledochotomy, while 30 (50%) patients had T-tube insertion.

Results: Among the total of 60 patients (primary closure: 30 and T-tube drainage: 30 patients), 10 (16%) were males, while 50 (84%) were female patients. The mean age was recorded as 45.9±13.9, and the mean hospital stay in days in both groups was 4.87±1.4 (T-tube: 5.24±1.50 and primary closure: 4.50±1.3). Complications developed in group A were a biliary leak in three (10%) patients, postoperative jaundice in two (6.5%) patients, wound infection in three (10%), intra-abdominal collection in three (10%) patients, and dislodgement of a tube in two (6.5%), while 17 (57%) patients had no complications developed.

Conclusion: If the duct is free of stones, as determined by preoperative choledochoscopy, primary closure of the common bile duct (CBD) is a safe, cost-effective procedure with less morbidity than regular T-tube drainage after open choledochotomy.

## Introduction

Around 10%-15% of people with gallbladder stones develop choledocholithiasis. Common bile duct (CBD) stones occur in 7%-15% of cholecystectomy patients [1]. It occurs more frequently in older age categories. It can result in a complete or partial blockage of the bile duct and present as pancreatitis or cholangitis due to gallstones. Endoscopic sphincterotomy and surgical exploration (choledochotomy) are the two methods used to treat CBD stones [2]. A supra-duodenal choledochotomy and the placement of a T-tube constitute the standard surgical management of CBD stones. Common bile ducts (CBDs) are typically closed over T-tubes, although this therapeutic approach has the potential for problems [3]. Following removal, complications such as bacteremia, tube dislodgment, obstruction/fracture, and bile leakage may occur. Before it can be removed, the patient might have to carry it for a few weeks. Each of them causes hospital stays to last longer. The T-tube drain may increase the risk of recurring stones because it acts as a foreign object around which bile pigments and salts may precipitate [4].

The primary CBD closure during choledochotomy is not a new phenomenon. More than a century ago, Thornton and Halstead wrote about primary duct closure following CBD exploration [5,6]. Numerous research articles published by various authors indicate the primary closure of the duct during CBD exploration.

Few surgeons today use laparoscopic surgery because it requires specialized training and tools for the management of CBD stones. Open CBD exploration is still a preferred approach in many institutions in underdeveloped countries since it has not yet been demonstrated that laparoscopic CBD exploration is superior to open CBD exploration for treating CBD stones [7]. Despite its clear benefits, main CBD closure is not frequently carried out. The preferred method for treating CBD stones in our hospital is still open CBD exploration and T-tube drainage. In this comparative study, the efficacy and safety of primary closure without T-tube drainage following open choledochotomy for CBD stones were evaluated.

## Materials and methods

Ethical approval has been obtained from the institutional ethical committee under reference number HMC/QAD/F/001191. This comparative study was carried out in General Surgery Department, Hayatabad Medical Complex Peshawar during a three-year period, from January 2018 to December 2021. The inclusion criteria for the study comprised patients with obstructive jaundice from choledocholithiasis as detected by magnetic resonance cholangiopancreatography (MRCP) and ultrasonography. All patients who were included had unsuccessful endoscopic retrograde cholangiopancreatography (ERCP) attempts at various sites. Participants excluded from the study consist of those who had pancreatitis, cholangitis, or malignancy and were younger than 20 or older than 75. Following exclusion, 60 patients in total were divided into two groups at random after receiving informed consent. Group A (30 patients) had T-tubes, and group B ( 30 patients) had primary closure. Routine tests were used to evaluate every patient. One hour before the onset of anesthesia, antibiotics were administered to all patients. To guarantee patency, stones were removed and then flushed with saline. The choledochoscope was used to confirm the duct's clearance before latex rubber T-tubes of the proper size (14-16 French size) were put into the CBD in group A patients, and interrupted Vicryl 2/0 was used to close the CBD primarily in group B patients without the use of a stent. Each patient received a single sub-hepatic closed suction drain at the conclusion of the treatment. Patients were allowed to walk the day following surgery and gradually resume oral intake as tolerated. If the closed suction drain produced a negligible output, it was removed before discharge. Patients were followed up for three months.

On the 14th postoperative day, group A patients underwent T-tube cholangiography as part of their initial follow-up as an outpatient. On the 16th postoperative day, it was removed once patency was determined. The two groups' hospital stays and postoperative complications were compared. To find any remaining stones, ultrasound or T-tube cholangiography was employed, and ultrasound was also used to evaluate any intra-abdominal collections that had formed after surgery. A wound that needs a partial or full wound opening is said to have a wound infection. Intra-abdominal collections were defined as those needing postoperative surgical or percutaneous drainage. Postoperative admission days were used to define hospital stay. SPSS 23.0 (Statistical Package for Social Sciences, IBM version 23.0, Chicago, USA) was used to enter and analyze the study's data.

## Results

There were 60 patients in total; 10 (16%) of them were men, and 50 (84%) were women. After the stone removal, 30 of these patients had primary closure of CBD, while the other 30 patients had T-tube drains installed. Patients in groups A and B had mean ages of 45.5±13.3 and 46.3±14.5, respectively. In the T-tube group, there were six (20%) males and 24 (80%) females, while in the primary closure group, there were four (13.33%) males and 26 (86.6%) females. Different clinical presentations of patients in both groups are shown in Table 1.

**Table 1:** Clinical presentation of patients

Presentation	Group A	Group B
Acute cholecystitis	5 (16.7%)	4 (13.3%)
Jaundice	8 (26.6%)	6 (20%)
Biliary colic	13 (43.4%)	17 (56.7%)
Dyspepsia	4 (13.3%)	3 (10%)

The postoperative hospital stay was significantly longer (5.24±1.50 days) in group A as compared to group B (4.6±1.3 days) (P=0.003). Complications developed in both groups were as follows: biliary leak in three (10%) vs one (3.5%) (p=0.710), postoperative jaundice in two (6.5%) vs one (3.5%) (p=0.901), wound infection in three (10%) vs two (6.5%) (p=0.661), and intra-abdominal collection in three (10%) vs two (6.5%) (p=0.901) (Table 2).

**Table 2:** Postoperative outcome

Outcome	Group A	Group B	P Value
Biliary leak	3 (10%)	1 (3.5%)	0.710
Jaundice	2 (6.5%)	1 (3.5%)	0.843
Wound infection	3 (10%)	2 (6.5%)	0.661
Intra-abdominal collection	3 (10%)	2 (6.5%)	0.901
Postoperative hospital stay	5.24±1.50	4.6±1.3	0.003

All wound infections needed wound opening and drainage along with antibiotics. Two patients (6.66%) had residual CBD stones in group A, while group B had no residual stones during follow-up. Except for one patient in group A who underwent ERCP and stenting for retained stones, all patients with biliary leakage were conservatively managed with blocked external biliary drainage.

## Discussion

For many years, open CBD exploration has been the primary method of treating CBD stones. At the moment, it is also regularly performed. In 1980, Courvoisier carried out the first CBD exploration, successfully removing CBD stones [8]. Endoscopic treatments are presently recognized as the first-line therapy for choledocholithiasis. ERCP was first introduced by McCune in 1968 [9]. But there is a dearth of equipment for these procedures in rural areas, even in the developed world. When ERCP is not an option, surgical exploration of the CBD is necessary. This can be accomplished either through laparoscopic or open CBD exploration. As a result, surgeons frequently have little choice but to carry on with open CBD exploration. In a study, Sheen-Chen and Chou revealed that ERCP was less effective than open surgery for removing CBD stones and was linked to a greater mortality rate [10]. Additionally, after endoscopic removal, the likelihood of CBD stones returning is doubled.

Open biliary surgery is a common choice among skilled surgeons, who successfully use this method. Although the tendency is in favor of primary closure, there is debate over the use of T-tubes against primary closure of the bile duct regardless of the technique. It is safe and related to a decreased complication risk to perform primary closure without a T-tube. However, certain hospitals continue to regularly insert T-tubes, particularly in developing countries like Pakistan [11]. The mean age in our study of both groups was comparable with a study done by Ambreen et al. [12]. Numerous studies revealed that, in comparison to T-tube drainage, primary CBD closure did not raise the risk of complications or death.

There was no statistically significant difference between the two groups in the incidence of bile leak, wound infection, jaundice, or intra-abdominal collection following the surgery in our study. De Roover et al. made similar findings as well [13]. In group A, out of three patients who had bile leak post-T-tube removal, only one needed ERCP and stenting for persistent bile leak due to missed CBD stones on the T-tube cholangiogram. The remaining two leaked resolved with percutaneous drainage only. Percutaneous drainage was a good treatment for the bile leak in group B patients. Based on the pus Culture and Sensitivity (C/S) report, wound infections in both groups were treated with antibiotics and wound irrigation. T-tube dislodgement in one patient on the 12th postoperative day had bile leak from the T-tube site for five days without any intra-abdominal collection.

One of the key elements of postoperative morbidity is an extended hospital stay. In our study, groups A and B had respective mean hospital stays of 5.2±1.50 and 4.5±1.3 days. In comparison to the primary closure group, the T-tube group had a longer hospital stay, according to multiple studies [14,15]. T-tube drainage following choledochotomy is still common in developing countries like Pakistan; however, there are not many research works contrasting it with primary CBD closure. To support the viability of primary CBD closure, additional research is needed because this is a single-institution study.

## Conclusions

The current study indicated that primary CBD closure with choledochotomy is safe and beneficial for the treatment of CBD stones while also enhancing patient care. Additionally, compared to patients using T-tubes, the morbidity and number of hospital admission days are much lower.
